# Peer Review of “Advancing Early Detection of Major Depressive Disorder Using Multisite Functional Magnetic Resonance Imaging Data: Comparative Analysis of AI Models”

**DOI:** 10.2196/76747

**Published:** 2025-07-15

**Authors:** Anonymous

**Keywords:** major depressive disorder, machine learning, functional MRI, early detection, artificial intelligence, psychiatry

*This is a peer-review report for “Advancing Early Detection of Major Depressive Disorder Using Multisite Functional Magnetic Resonance Imaging Data: Comparative Analysis of AI Models.”*

## Round 1 Review

### Specific Comments

#### Major Comments

1. This paper [1] provides sufficient information about major depressive disorder and the potential of artificial intelligence (AI); it could benefit from a more detailed comparison with the existing literature. How does the present study build on or extend previous work? Additional details on why previous AI studies have not focused on early detection could help contextualize the research gap you are addressing.

#### Minor Comments

2. It’s also important to emphasize that AI should complement, rather than replace, clinical expertise.

## Abbreviations

AI: artificial intelligence
